# Spread from the Sink to the Patient: *In Situ* Study Using Green Fluorescent Protein (GFP)-Expressing Escherichia coli To Model Bacterial Dispersion from Hand-Washing Sink-Trap Reservoirs

**DOI:** 10.1128/AEM.03327-16

**Published:** 2017-03-31

**Authors:** Shireen Kotay, Weidong Chai, William Guilford, Katie Barry, Amy J. Mathers

**Affiliations:** a Division of Infectious Diseases and International Health, Department of Medicine, University of Virginia Health System, Charlottesville, Virginia, USA; b Department of Biomedical Engineering, University of Virginia, Charlottesville, Virginia, USA; c Clinical Microbiology, Department of Pathology, University of Virginia Health System, Charlottesville, Virginia, USA; University of Manchester

**Keywords:** antibiotic resistance, biofilms, dispersion, hand washing, hospital infections, premise plumbing, sink, transmission, wastewater

## Abstract

There have been an increasing number of reports implicating Gammaproteobacteria as often carrying genes of drug resistance from colonized sink traps to vulnerable hospitalized patients. However, the mechanism of transmission from the wastewater of the sink P-trap to patients remains poorly understood. Herein we report the use of a designated hand-washing sink lab gallery to model dispersion of green fluorescent protein (GFP)-expressing Escherichia coli from sink wastewater to the surrounding environment. We found no dispersion of GFP-expressing E. coli directly from the P-trap to the sink basin or surrounding countertop with coincident water flow from a faucet. However, when the GFP-expressing E. coli cells were allowed to mature in the P-trap under conditions similar to those in a hospital environment, a GFP-expressing E. coli-containing putative biofilm extended upward over 7 days to reach the strainer. This subsequently resulted in droplet dispersion to the surrounding areas (<30 in.) during faucet operation. We also demonstrated that P-trap colonization could occur by retrograde transmission along a common pipe. We postulate that the organisms mobilize up to the strainer from the P-trap, resulting in droplet dispersion rather than dispersion directly from the P-trap. This work helps to further define the mode of transmission of bacteria from a P-trap reservoir to a vulnerable hospitalized patient.

**IMPORTANCE** Many recent reports demonstrate that sink drain pipes become colonized with highly consequential multidrug-resistant bacteria, which then results in hospital-acquired infections. However, the mechanism of dispersal of bacteria from the sink to patients has not been fully elucidated. Through establishment of a unique sink gallery, this work found that a staged mode of transmission involving biofilm growth from the lower pipe to the sink strainer and subsequent splatter to the bowl and surrounding area occurs rather than splatter directly from the water in the lower pipe. We have also demonstrated that bacterial transmission can occur via connections in wastewater plumbing to neighboring sinks. This work helps to more clearly define the mechanism and risk of transmission from a wastewater source to hospitalized patients in a world with increasingly antibiotic-resistant bacteria that can thrive in wastewater environments and cause infections in vulnerable patients.

## INTRODUCTION

Despite early reports (1–5), the premise that hand-wash sink traps can act as reservoirs of bacteria that cause nosocomial infections has been frequently overlooked. There has recently been an alarming increase in sink-related outbreaks worldwide, with many reports establishing an observational link (6–13). A sink often operates as an open conduit to wastewater in a patient care area that is often in the same room as the patient.

Health care establishments often invest in desperate interventions to deal with nosocomial outbreaks. The preferred method for addressing most of the environment-related transmission is to employ enhanced cleaning using chemical and physical agents (14, 15). Unfortunately, routine approaches are inefficient in completely eliminating drug-resistant Gammaproteobacteria in an inaccessible microbiologically active area such as a sink trap (6, 16–20). The wet, humid, and relatively protected environment in a sink trap favors the formation of rich stable microbial communities (16, 21, 22). These communities will be exposed to liquids and waste that are discarded in a sink and may include antimicrobials, discarded beverages, soap, presumably pathogenic bacteria from health care workers' hands, and other items. In short, sink traps could serve as a breeding ground for opportunistic and highly antimicrobial-resistant bacteria that cannot be easily cleaned or removed (23–28).

There are many reports of a genetic association between pathogens found in sink traps and those found in patients (29, 30). However, surprisingly little work has been done to understand the microscale transmission dynamics. It was previously demonstrated using a suspension of fluorescent particles (Glo Germ; Glo Germ Co., Moab, UT) that material injected into the P-trap gets dispersed around a hand-washing sink (6). This result however has not been replicated hitherto in the follow-up studies. Dispersion has never been investigated with living organisms. Ultimately, many details remain unaddressed surrounding the spread of Enterobacteriaceae in sink-trap wastewater systems: (i) can organisms grow retrograde from the P-trap water to the sink strainer, (ii) can organisms spread from one sink to another along the internal surfaces of pipes with shared drainage systems, and (iii) which portion of a colonized drain pipe results in dispersion into the sink bowl during a hand-washing event? We aim to better understand the dispersion dynamics of Gammaproteobacteria living in the wastewater of a sink strainer and P-trap into an area where patients and health care workers could be exposed. To study this dynamic, we used a surrogate organism that could be easily tracked while remaining in the Enterobacteriaceae family, where some of the most concerning threats in antimicrobial resistance are developing (30).

## RESULTS

### Growth and colonization of GFP-expressing E. coli in the P-trap.

In the first 14 days following the installation of the P-trap with established green fluorescent protein (GFP)-expressing Escherichia coli and just water running from the faucet, GFP-expressing E. coli was not detected in the tailpipe beyond 1.5 in. above the liquid level in the P-trap. GFP-expressing E. coli, however, was found to be viable in the P-trap without any nutrients added. A nutrient regimen was then instituted to understand the influence of nutrients on mobility and upward growth. The addition of tryptic soy broth (TSB) promoted GFP-expressing E. coli growth as early as day 1, with growth observed in the tailpipe 2 in. above the liquid surface in the P-trap (Table 1). On day 7, the strainer (∼8 in. above the liquid in the P-trap) was found to be colonized with GFP-expressing E. coli. This translates to an average growth rate of 1 in./day along the length of the tailpipe with the addition of nutrients and without faucet operation. GFP-expressing E. coli was not detected in the faucet water.

**TABLE 1:** Growth in the tailpipe connected to the P-trap colonized with GFP-expressing E. coli biofilm

Sampling area	Presence of GFP-expressing E. coli on day^*a*^:							
	0	1	2	3	4	5	6	7
Strainer 8 in. above P-trap water	**−**	**−**	**−**	**−**	**−**	**−**	**−**	**+**
Tailpipe								
6 in. above P-trap water	**−**	**−**	**−**	**−**	**+**	**+**	**+**	**+**
4 in. above P-trap water	**−**	**−**	**−**	**+**	**+**	**+**	**+**	**+**
2 in. above P-trap water	**−**	**+**	**+**	**+**	**+**	**+**	**+**	**+**
P-trap	**+**	**+**	**+**	**+**	**+**	**+**	**+**	**+**

a “−” and “+” denote the absence and presence of GFP-expressing E. coli, respectively.

### Sink-to-sink transmission of bacteria.

**FIG 1:**
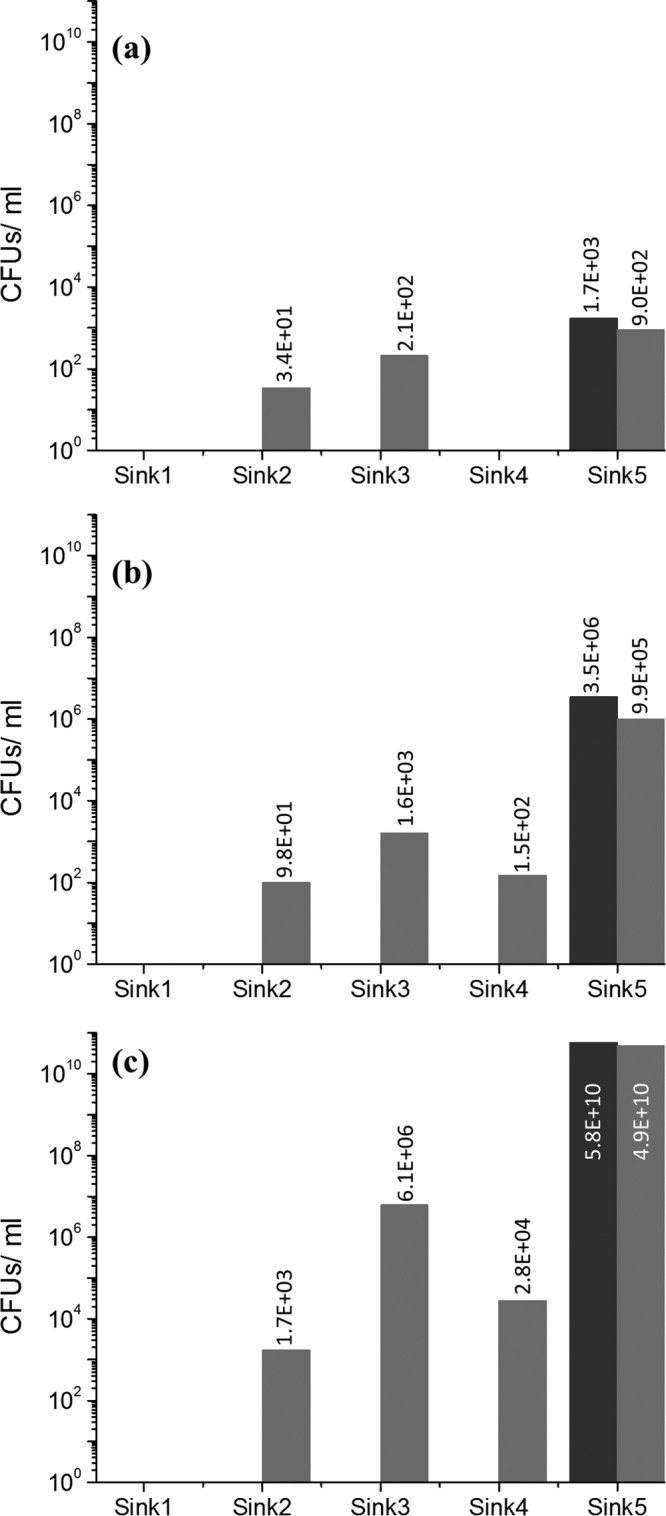
GFP-expressing E. coli detected in the P-traps attached to each of the sinks on day 0 (black bars) and day 7 (gray bars) using (a) 10^3^, (b) 10^6^, and (c) 10^10^ CFU/ml as the starting inoculum concentrations in sink 5.

In these experiments, a flanking sink (sink 5) was the only P-trap inoculated with GFP-expressing E. coli and therefore was the sole source for transmission to the connected sinks. Starting with a lower inoculum concentration (10^3^ CFU/ml) in sink 5, on day 7, GFP-expressing E. coli was detected in the sink 2 and sink 3 P-traps (Fig. 1a). With inoculum concentrations of 10^6^ CFU/ml and >10^10^ CFU/ml in sink 5, all of the sink P-traps in the sink gallery with the exception of sink 1 were found to be colonized with GFP-expressing E. coli after 7 days (Fig. 1b and c). Faucet water and aerators tested negative for GFP-expressing E. coli. Irrespective of the starting inoculum concentration, on day 7 the highest level of colonization was recorded in the sink 3 P-trap. After day 7, when the nutrient regimen (described previously) was followed for an additional 7 days in each of the sinks in the sink gallery with an inoculum concentration of >10^10^ CFU/ml, GFP-expressing E. coli was detected in the strainers of sinks 2 and 3 on day 14. This finding validated the upward growth and growth rate in the tailpipe when nutrients were added. Nonfluorescent colonies were occasionally observed in the P-trap water samples collected from the sinks, which were subsequently identified to be Pseudomonas sp. or Stenotrophomonas maltophilia, and fluorescent colonies were confirmed to be E. coli.

### Dispersion of microspheres from sinks.

In the first dispersion experiment, when fluorescent microspheres were inoculated into the offset drain tailpiece only 4 in. below the strainer, no microspheres were detected on the polyester sheets placed on the counter space.

**FIG 2:**
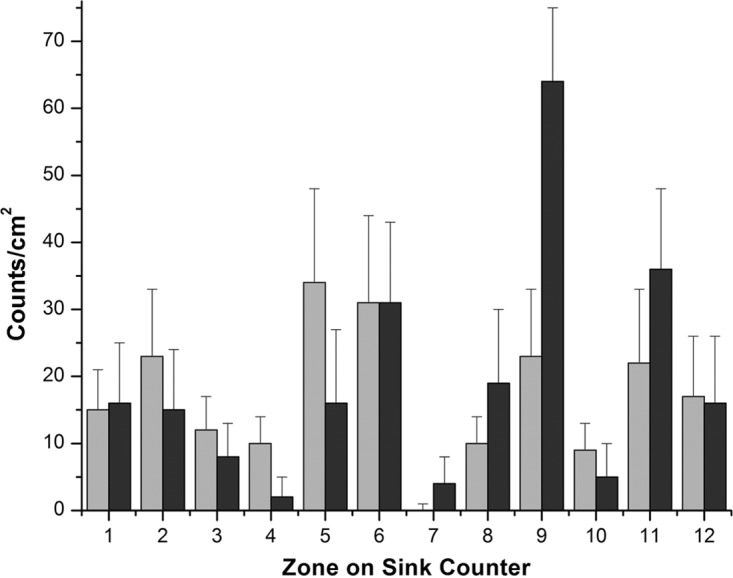
Dispersion of microspheres (gray bars) and GFP-expressing E. coli (black bars) on the area surrounding the sink when the sink bowl was coated. The *x* axis represents the designated zones of the sink counter.

However, when the sink bowl was coated with the microspheres, polyester sheets overlaid on the counter space captured the dispersed microspheres caused by the faucet operation. Dispersion was observed on almost all zones of the sink counter space (Fig. 2). Relatively higher levels of dispersion were observed along the major and minor axes of the elliptical sink bowl (zones 2, 5, 6, 9, 11, and 12). Anterior corners of the sink counter space (zones 4 and 7), which were most distant from the impact of water in the sink bowl, received the lowest dispersion.

### Dispersion of GFP-expressing E. coli from sinks.

Initially the P-trap alone was inoculated with GFP-expressing E. coli and carefully installed, keeping the tailpipe and strainer free of GFP-expressing E. coli before operating the faucets. No fluorescent CFU were observed on the plates placed on the counter or attached to the bowl surface after faucet operation. Similarly, no fluorescent CFU were detected when GFP-expressing E. coli was inoculated into the offset drain tailpiece only 4 in. below the strainer. Interestingly, when there was conspicuous water backup over the strainer as a result of a higher water flow rate from the faucet than the drainage rate from the P-trap, dispersal was detected on the plates attached to the bowl surface.

The dispersion pattern recorded when the sink bowl was coated with GFP-expressing E. coli was comparable to the pattern recorded when the sink bowl was coated with fluorescent microspheres (Fig. 2). Dispersion was significantly higher along the axes (zones 6, 9, 11, and 12) and lower at the corners of the sink counter space (zones 4, 7, and 10).

**FIG 3:**
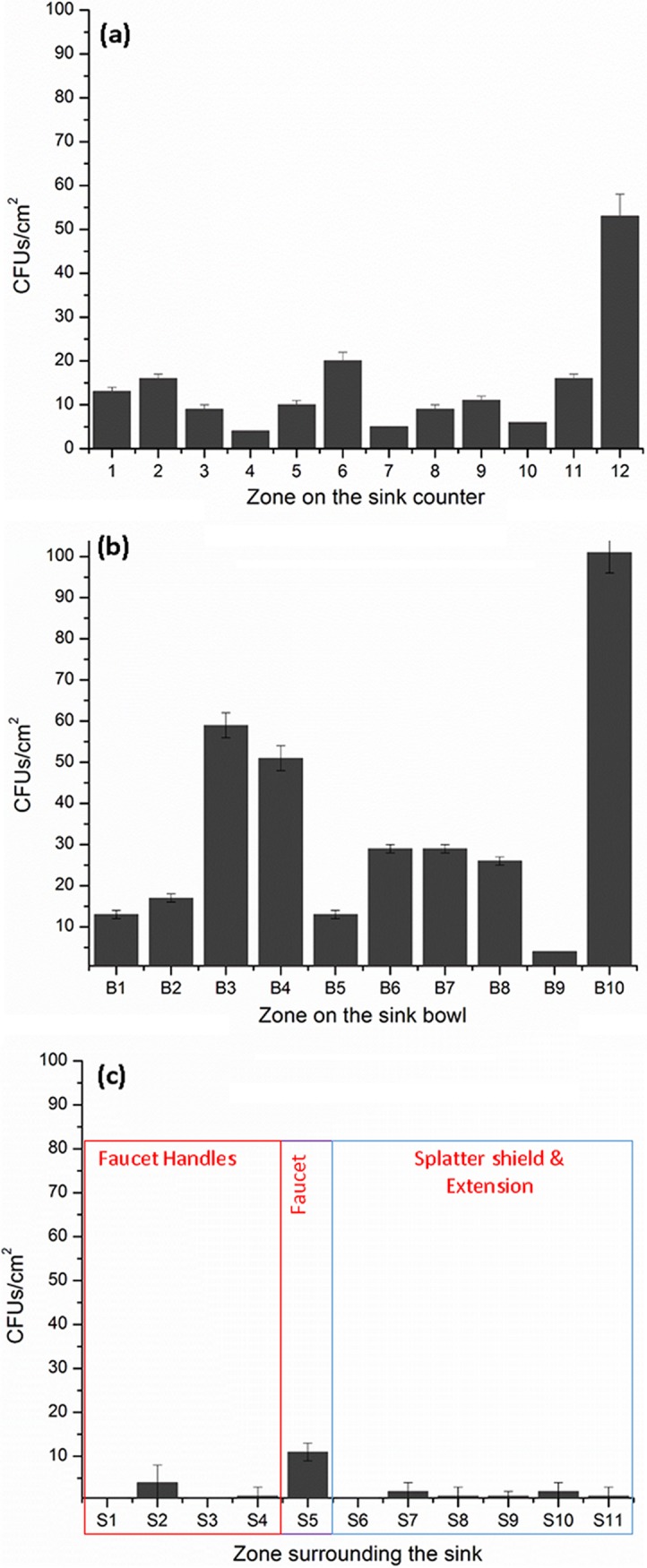
Dispersion of GFP-expressing E. coli on the area surrounding the sink when the strainer, tailpipe, and P-trap were colonized. (a) Sink counter; (b) sink bowl; (c) other surrounding areas. The *x* axis represents the designated zones of the sink counter.

In contrast, dispersion of GFP-expressing E. coli caused by the faucet operation was much more extensive when the strainer was allowed to be colonized with GFP-expressing E. coli prior to the dispersion experiment. In addition to the sink counter space, we measured dispersion to the sink bowl, faucet, faucet handles, splatter shields, and the extended counter surface. Dispersion of GFP-expressing E. coli was highest on the plates attached to the sink bowl (Fig. 3b). Further, dispersion was greater along the minor axis of the sink bowl (Fig. 3b, zones B3, B4, and B10) than along the major axis of the sink bowl, associated with a shorter distance from the strike point of the faucet water to the bowl along this axis. The next highest CFU count from the dispersal was recorded on the counter area near the faucets (Fig. 3a, zones 12 and 11). A similar pattern of higher dispersion near the faucets and lower dispersion at the corners of the counter space (Fig. 3a, zones 4, 7, and 10) was also observed using microspheres. Dispersion was also recorded in other zones of the counter space, on the Plexiglas splatter shields, faucets, faucet handles, and extended surface (Fig. 3c). There were no GFP-expressing E. coli CFU recorded on plates placed beyond 30 in. from the strainer, demarcating the range of dispersion under these experimental conditions.

Table 2 gives a summary of the total distribution loads recorded using fluorescent microspheres and GFP-expressing E. coli across each experiment. The loads of dispersion on the sink counter were comparable when the sink bowl was coated with microspheres or GFP-expressing E. coli before the faucet operation. Although the dispersion load on the sink counter was lower when the sink strainer was colonized, it is interesting to note that the sink bowl received the highest dispersion.

**TABLE 2:** Comparison of dispersion loads across different experiments

Dispersion expt	Dispersion load			
	Sink counter (>30 in.)	Sink bowl	Faucets and faucet handles	Splatter shields
Microspheres (microspheres/cm^2^)				
Offset drain inoculated with microspheres	0	NA^*a*^	NA	NA
Sink bowl coated with microspheres	206 ± 10	NA	NA	NA
GFP-expressing E. coli (CFU/cm^2^)				
P-trap inoculated with GFP-expressing E. coli	0	0	0	0
Offset drain inoculated with GFP-expressing E. coli	0	NA	NA	NA
Sink bowl coated with GFP-expressing E. coli	232 ± 17	NA	NA	NA
Strainer colonized with GFP-expressing E. coli	171 ± 15	342 ± 17	17 ± 3	3 ± 1

a NA, not applicable.

## DISCUSSION

To mimic dispersion in a hospital setting, we first investigated whether GFP-expressing E. coli would establish consistent colonization in a sink trap as many other Gammaproteobacteria implicated in nosocomial outbreaks have done (6, 28). Many recent reports demonstrate that P-traps become colonized with highly consequential Gammaproteobacteria, which then results in nosocomial transmission (29, 31, 32). The retained water in a sink P-trap is present to provide a water barrier to prevent off-gassing of sewer smell, but it may inadvertently provide favorable conditions for pathogenic and opportunistic antibiotic-resistant microorganisms to survive and develop resilient biofilms (3, 33). However, the mechanism of dispersal of the bacteria in the P-trap to patients or the surrounding health care area had not been fully elucidated. We began with the hypothesis that the bacteria originate from the P-trap via droplet creation when the water from the faucet hits the P-trap water, thus contaminating the sink bowl and the surrounding area. The finding supporting this theory had been previously reported using Glo Germ particles (6). However, in the present study with careful attention to avoid strainer and tail piece contamination, the dispersal directly from the sink P-trap with either microspheres or GFP-expressing E. coli could not be reproduced as previously reported (6).

Rather this work demonstrates a different, more staged mode of transmission from a P-trap reservoir to the sink and surrounding environment. GFP-expressing E. coli in the P-trap alone was sustained for 14 days but did not grow or mobilize up the tailpipe to the strainer with just intermittent water exposure. However, when nutrients were subsequently added to the system, the organisms rapidly grew up the tailpipe to the strainer at approximately an inch per day. In a real-world setting, motility of bacteria inside the tailpipe is restricted to relatively sporadic and brief wetting events in which swimming is an opportunity to colonize new surfaces. It is assumed that once established, the biofilm promotes the upward growth of GFP-expressing E. coli in the tailpipe at an accelerated rate. The nutrient regimen that promoted colonization in our model reflects our and others' observations of items commonly disposed of in hospital sinks (intravenous fluids, feeding supplements, and leftover beverages) (5, 32).

**FIG 4:**
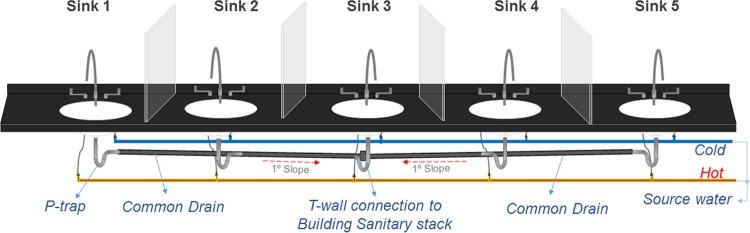
Layout of the sink gallery comprising the 5 sink modules and the associated plumbing.

Transmission of bacteria between sinks via a common pipe was a key finding in this study as this highlights the concept that premise plumbing may be a more continuous system with shared microbiology than a single isolated sink. The sink gallery used in this study provided a unique *in situ* advantage to investigate sink-to-sink transmission of bacteria through common drains. The two possible mechanisms for P-trap strainers becoming colonized are seeding of organisms from above and retrograde spread of organisms along common pipes in a hospital wastewater infrastructure. Here we demonstrate that it is possible for GFP-expressing E. coli to contaminate adjacent P-traps with just time and water given a standard U.S. code piping rise of 0.25 ft/ft. Sink-to-sink or retrograde transmission may explain the recurrence of pathogen colonization following intervention strategies like disinfection or replacement of plumbing (23). Sink 3 was lowest on the slope in the drain line (see Fig. 4) with arguably the most opportunity for reflux and retrograde wetting. Sink 1, on the other hand, was farthest away from the source (sink 5), and its P-trap had the greatest incline in the drain line connecting the sinks, which could perhaps contribute to the reasons there was no GFP-expressing E. coli colonization detected in it after 7 days. There has been more investigation about microbiologic dynamics of infectious viral particles such as those of severe acute respiratory syndrome (SARS) and Ebola viruses through premise plumbing systems (34–36). However, the microbiology, sustainability, and dynamics might be very different, although the backflow and inoculation issues could have some parallels when comparing viruses to bacteria. As Enterobacteriaceae can either multiply or remain viable for long periods of time in biofilms coating the interior of P-traps and the connected plumbing, it may not be sustainable to target any intervention limited to a single isolated sink as a source of a particular pathogen.

Data from different dispersal experiments suggest that although P-traps can act as the source or the reservoir of pathogens, the physical presence of the organism in the sink bowl or colonization of the strainer is necessary for the dispersal to occur. Colonization of strainers or drains reported in earlier studies (7, 10, 13, 24, 37) was perhaps a result of ascending biofilm growth from the P-trap to the strainer or introduction through contaminated fluids. Many of the studies used swab samples, which likely sampled the strainer rather than P-trap water (17, 20). Once the strainer was colonized, the water from the faucet resulted in GFP-expressing E. coli dispersion in the bowl and to the surrounding surfaces of up to 30 in. The range of dispersal recorded in this study was comparable to that reported earlier (6). Greater dispersal near the faucet may be attributed to the specific designs of the sink bowl and faucet in this study, which determine the contact angle of water impact. This is an important finding since many sinks in hospitals are similar in design, with faucet handles representing a high-touch surface for the sink users (38). It can also be concluded from the dispersion experiments that secondary and successive dispersals would likely increase the degree and the scope of dispersion.

There are several limitations to this work. First the use of similar sink bowls across these sinks only examines the dispersion pattern of this particular sink design. Similarly the sink-to-sink transmission may not be applicable to all wastewater plumbing systems as the fixtures on the pipe are very close together, unlike most layouts in health care settings. However, we speculate that transmission could occur on larger systems over greater time scales, especially if heavy nutrient and contamination loads were also included. GFP-expressing E. coli is a laboratory surrogate, and the putative biofilms established in the short time frame of our experiments are unlikely to be as complex or stable as biofilms developed in a hospital wastewater system over many years. However, to address the monomicrobial dominance of the GFP-expressing E. coli added to the system, we kept the system open, and other environmental organisms were able to cocolonize in an attempt to mimic the hospital system. Another limitation was the need to add nutrients to the drain to ensure rapid and robust colonization. We are not clear how widespread the practice of disposing of dextrose-containing intravenous fluids or leftover beverages in the hand-wash sinks is; however, we have observed this practice, and anecdotally it appears to be relatively common in the United States. We also did not completely characterize the droplet sizes, nor do we demonstrate air sampling to understand if the dispersion is only droplet or if there are also aerosols that contain GFP-expressing E. coli. This would require additional testing and is planned as future work.

In summary, this work for the first time better models the mechanisms of spread of multidrug-resistant pathogens arising from the sink drain and infecting patients. Droplet dispersion from the P-trap does not happen directly. Rather it is a multistage process: dispersal originates from the strainer and/or the bowl after growth of the biofilm up from the microbial reservoir of the P-trap. We also demonstrate sink-to-sink transmission via a common sanitary pipe. This work could have implications for patient safety, infection control, and interventions as well as the design of future hospital plumbing systems to eliminate this mode of transmission to vulnerable hospitalized patients.

## MATERIALS AND METHODS

### Sink gallery design.

A dedicated sink gallery was set up to simulate hospital hand-washing sinks. The gallery was comprised of five sink modules assembled next to each other (Fig. 4). The five hand-wash sink stations were identical in bowl designs and dimensions and were modeled from the most common intensive care unit hand-washing sink type in the acute care hospital at the University of Virginia Medical Center. Partitions made of 24-in.-high Plexiglas sheet were installed between the sinks to prevent splatter and cross contamination. Each sink module was built with Corian integrated sink/countertops without an overflow and fitted with an 8-in. centerset 2-handle gooseneck faucet (Elkay, Oak Brook, IL). The drain line under each sink was comprised of a flat-top fixed strainer (drain size, 2 in. by 3 in.), 17-gauge (1.47-mm-thick) 8- to 10-in.-long tailpipe, P-trap, and trap arms of 0.25-in. outside diameter (o.d.) (Dearborn Brass-Oatey, Cleveland, OH). All of the fixtures were made of brass with chrome plating. Each of the sink P-traps was connected to a 3-in. common cast-iron pipe sloping into a T-joint leading into the building sanitary line located behind sink 3 (Fig. 4).

### Inoculation, growth, and establishment of GFP-expressing E. coli in sink P-traps.

For the GFP-expressing E. coli strain (ATCC 25922GFP), the green fluorescent protein (GFP) gene is contained on a plasmid that also contains an ampicillin resistance gene. A single isolated colony of GFP-expressing E. coli grown from a −80°C stock was inoculated into 5 ml tryptic soy broth (TSB) (Becton, Dickinson and Company, Sparks, MD) containing 100 μg/ml ampicillin (ATCC medium 2855). The inoculum concentration and method varied for each experiment. For establishment of GFP-expressing E. coli in sink P-traps, new autoclaved P-traps were filled with 100 ml 0.1× TSB and inoculated with ∼10^3^ CFU/ml GFP-expressing E. coli. Following inoculation, both ends of the P-traps were covered with perforated Parafilm (Bemis, Inc., Oshkosh, WI) and allowed to incubate at room temperature (22 ± 2°C) for 14 days to facilitate adherent bacterial growth. The medium in the P-trap was decanted and replaced with fresh 0.1× TSB every 48 h. An aliquot of decanted medium and a swab sample from the inner surface of the P-trap were plated on tryptic soy agar (Becton, Dickinson and Company, Sparks, MD) plates containing 100 μg/ml ampicillin (TSA) to monitor the growth of GFP-expressing E. coli in the P-traps. TSA plates were incubated overnight at 37°C, and CFU fluorescing under UV light were enumerated. All preparatory culturing of GFP-expressing E. coli took place in a separate room from the sink gallery to avoid unintentional contamination.

### Installation of P-traps colonized with GFP-expressing E.coli.

**FIG 5:**
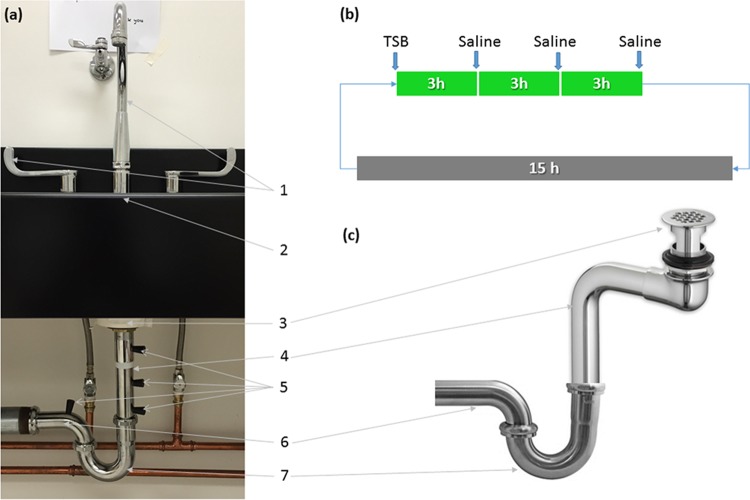
(a) Parts of the sink drain line: 1, faucet and handles; 2, sink counter; 3, strainer; 4, tailpipe; 5, sampling ports; 6, trap arm; 7, P-trap. (b and c) Schematic of the nutrient regimen (b) and offset drain tailpiece used for dispersion experiments (c).

After the 14-day incubation, P-traps were fastened into the plumbing of the sinks (Fig. 5a). The remainder of the drain line was either autoclaved (strainer, tailpipe, and trap arms) prior to installation or surface disinfected (sink bowl, countertop, and faucets) with Caviwipes-1 (Meterx Research, Romulus, MI), maintaining at least 1 min of contact time. After the P-trap was installed, a daily regimen was followed in which 25 ml of TSB followed by 25 ml of 0.9% NaCl solution (saline) were added in the ratio 1:3 via the strainer (Fig. 5b) to mimic the potential nutrient exposure in the hospital.

### Sampling and enumeration of GFP-expressing E. coli.

To monitor the growth of GFP-expressing E. coli in the plumbing, sampling ports were drilled along the length of the tailpiece (between the P-trap and the strainer) and the trap arm (between the P-trap and the common line). These holes were fitted with size 00 silicone stoppers (Cole-Parmer, Vernon Hills, IL) (Fig. 5a). Sterile cotton swabs (Covidien, Mansfield, MA) presoaked in saline were inserted through these sampling ports, and samples were collected by turning the swab in a circular motion on the inner surface (∼20 cm^2^) of the tailpipes. Sample swabs were pulse-vortexed in 3 ml saline, and serial dilutions were plated on TSA. The strainer, faucet aerator, and bowl surface were sampled with presoaked swabs and processed as described earlier.

### Sink-to-sink transmission of bacteria.

To investigate sink-to-sink transmission of bacteria, a distal sink (sink 5) (Fig. 4) was fitted with a P-trap inoculated with GFP-expressing E. coli. The effects of different inoculum concentrations of GFP-expressing E. coli—10^3^, 10^6^, and >10^10^ CFU/ml (colonized for 14 days)—were investigated. Identification to the species level of fluorescent and nonfluorescent colonies identified from mixed pipe cultures was performed using a matrix-assisted laser desorption-ionization (MALDI)–time of flight (MALDI-TOF) mass spectrometer (Vitek-MS; bioMérieux, Durham, NC). The wastewater paths of sinks 1 to 4 were either autoclaved (strainer, tailpipe, P-traps, and trap arms) prior to installation or surface disinfected (sink bowl, countertop, and faucets) with Caviwipes-1 (Meterx Research, Romulus, MI). Faucets on each of the five sinks were turned on simultaneously for 1 min, supplying water at a flow rate of 8 liters/min, once every 24 h for 7 days. No additional feed to any of the sinks was added during this period of 7 days. On days 0 and 7, P-traps on each of the five sinks were unfastened, and swab samples from the P-trap were collected and processed as described earlier.

### Dispersion measured using fluorescent microspheres.

Fluoresbrite YO carboxylate microspheres (Polysciences, Inc.) which had a 1-μm diameter and maximum excitation and emission of 529 nm and 546 nm, respectively, were used as a tracer in the preliminary experiments to understand droplet dispersion from the hand-wash sinks.

**FIG 6:**
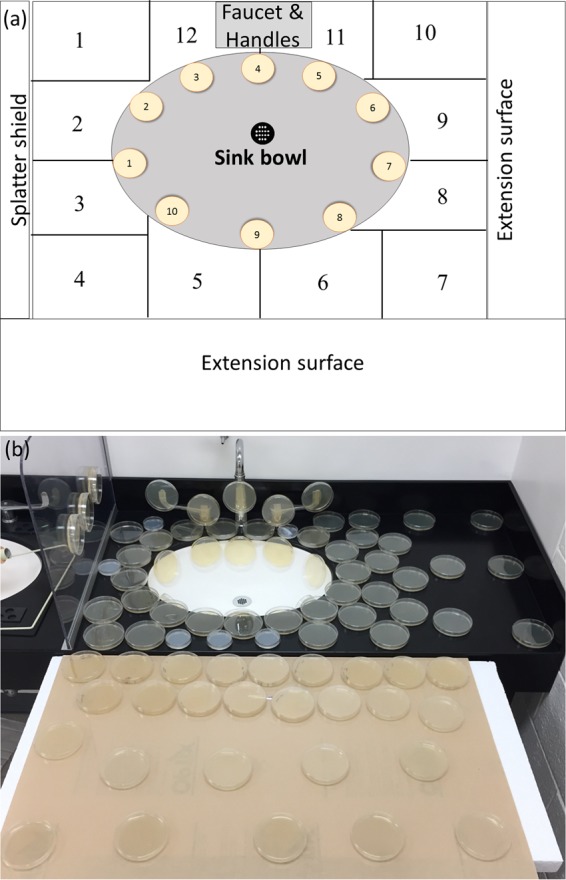
(a) Layout of the zones of the sink counter, bowl, and extension surface designated to monitor droplet dispersion and (b) layout of the TSA plates used for GFP-expressing E. coli droplet dispersion on the surfaces surrounding the sink.

To test whether microspheres could be dispersed from below the sink strainer, a 1-ml suspension of microspheres (∼10^10^ particles) was injected through a strainer attached to a Hert 4.5-in. offset drain tailpiece typically used for wheelchair-accessible sinks (American Standard, model 7723018.002) (Fig. 5c). The vertical distance between the strainer and microsphere suspension injected into the tailpipe was ∼4 in. Counter space around the sink bowl was thoroughly wiped with alcohol wipes (Covidien Webcol 6818; Kendall), and polyester sheets precut to appropriate shapes were placed on the counter to cover the entire sink counter and labeled according to position (Fig. 6a). The faucet was turned on for 5 min at a water flow rate of 1.8 to 3.0 liters/min. Polyester sheets were harvested and immediately analyzed using a ChemiDoc MP system (Bio-Rad Laboratories, Inc.) with an exposure time of 5 s. Fluorescent microspheres were enumerated from the digital micrographs using the Image Lab Software (Bio-Rad Laboratories, Inc.).

To test whether microspheres could be dispersed from the surface of the sink bowl, the sink bowl was evenly coated with a 20-ml microsphere suspension (∼10^10^ particles/ml) using a disposable swab (Sage Products, Inc., Cary, IL), and the dispersion experiment was repeated following the protocol described above. To ascertain there was no nonspecific background fluorescence in the sink and/or the water from the faucet, a control using the same protocol but without the fluorescent microspheres was performed before each experiment.

### Dispersion measured using GFP-expressing E.coli.

Dispersion using GFP-expressing E. coli was investigated in three experiments. To test whether live organisms in the P-trap could be dispersed by running water, ∼10^10^ CFU/ml GFP-expressing E. coli in saline was added to an autoclaved P-trap and fitted into the drain line that was preautoclaved (strainer, tailpipe, and trap arms). Similarly, to test whether live organisms could be dispersed from the tailpieces of wheelchair-accessible sinks, a suspension of ∼10^10^ CFU/ml GFP-expressing E. coli was added with a syringe through the strainer into the Hert 4.5-ft offset drain tailpiece (Fig. 5c). Just as in the microsphere dispersion experiment, the vertical distance between the strainer and GFP-expressing E. coli suspension injected into the tailpipe was ∼4 in.

We next tested whether live organisms from the surface of the sink bowl could be dispersed by running water. The sink bowl surface was evenly coated with an approximately 20-ml suspension of 10^10^ CFU/ml GFP-expressing E. coli.

Finally, to mimic all of these conditions, a P-trap colonized with GFP-expressing E. coli (for 14 days) was installed, and a nutrient regimen (Fig. 5b) was followed for 14 days to intentionally promote the GFP-expressing E. coli colonization in the attached tailpipe and strainer. On day 15, the dispersion experiment was performed.

Before each of the GFP-expressing E. coli dispersion experiments, the counter space was thoroughly disinfected with Caviwipes-1. TSA plates were then positioned on the sink counter surrounding the bowl and an extension platform (Fig. 6b). Additional plates were attached to the sink bowl, faucets, Plexiglas partitions, and faucet handles using adhesive tape. TSA plates were also placed 3 m away from the sink as negative controls. The faucet was turned on for 5 min with a water flow rate of 1.8 to 3.0 liters/min. Lids of the TSA plates were removed only during faucet operation. Swab samples from the faucet aerators before and after operation were collected and plated on TSA. Prior to each dispersion experiment, 50 ml water from the faucet was also collected, and aliquots were plated to assess for the presence of GFP-expressing E. coli in source water and ensure cross contamination of GFP-expressing E. coli had not occurred. A control dispersion experiment was also performed using the same protocol prior to GFP-expressing E. coli inoculation in each case. Dispersion per defined area (CFU per square centimeter) was deduced by dividing the CFU counts in the TSA plate by the surface area of the TSA plate.
